# Metal–Organic Framework Multizyme Colloids with Joint Antioxidant and Protease Function

**DOI:** 10.1021/acs.langmuir.5c06847

**Published:** 2026-07-07

**Authors:** Laila Noureen, Dániel Viczián, Gergely F. Samu, Imre Szenti, Bojana Katana, Tamás Szabó, Viktoria Hornok, Zoltán Kónya, Istvan Szilagyi

**Affiliations:** ‡ MTA-SZTE Momentum Biocolloids Research Group, Interdisciplinary Centre of Excellence, 37442 University of Szeged, Szeged 6720, Hungary; ⧧ Department of Molecular and Analytical Chemistry, 37442 University of Szeged, Szeged 6720, Hungary; § Department of Applied and Environmental Chemistry, 37442 University of Szeged, Szeged 6720, Hungary; † Institute of Condensed Matter and Nanosciences - Bio and Soft Matter, 83415 Université catholique de Louvain, Louvain-la-Neuve 1348, Belgium; £ Department of Physical Chemistry and Materials Science, 37442 University of Szeged, Szeged 6720, Hungary

## Abstract

Metal–organic framework (MOF) nanozymes with multiple biocatalytic functions remain scarce, and their colloidal behavior, critical for applications in liquid media, has received limited attention. Here, we report fine aqueous dispersions of a bimetallic CuZr-MOF nanozyme composed of small particles exhibiting a triple enzyme-mimicking activity. Comprehensive microscopy, spectroscopy, and diffraction analyses revealed the structural features of the CuZr-MOF. The surface charges, hydrodynamic sizes, and aggregation rates were systematically investigated in monovalent electrolyte solutions, enabling the identification of well-defined colloidal stability regimes across a broad range of conditions. Pronounced ion specific effects were observed at the solid–liquid interface, and a combined DLVO-Hofmeister description successfully captured the origin of interparticle forces. The CuZr-MOF displayed superoxide dismutase (SOD), horseradish peroxidase (HRP), and, for the first time, protease-like activity, demonstrating a unique combination of antioxidant and hydrolytic functions within a single MOF nanoparticle. The integrated assessment of enzymatic and colloidal properties highlights the strong potential of multifunctional MOF nanozymes for advanced biocatalytic, sensing, and therapeutic applications in liquid-phase environments.

## Graphical abstract

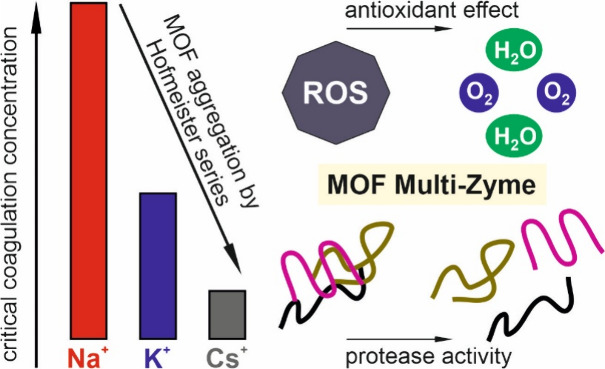

## Introduction

The high cost, strict handling requirements, and limited stability of natural enzymes under environmental stress create a persistent need for robust substitutes such as enzyme-mimicking nanomaterials, commonly referred to as nanozymes.^1–3^ Since the seminal 2007 report demonstrating the peroxidase (POD)-like activity of Fe_3_O_4_ nanoparticles, nanozyme systems have expanded rapidly.^4^ They provide a platform that could overcome the existing shortcomings in natural enzymes by offering facile synthetic routes, improved catalytic efficiency, and resistance against environmental conditions.^5–8^ Despite the large number of reports on various nanozyme materials, several important problems remain to be tackled in the field. For instance, mimics of single enzymes have been reported several times,^9–11^ but multifunctionality, i.e., mimicking enzymes of different functions with the same nanozyme, remains challenging.^12–14^ In parallel, colloidal properties such as surface charge, hydrodynamic size, and interface structure, which critically influence nanozyme activity and stability in liquid media, have received far less attention despite their key role in determining overall biocatalytic performance.^15,16^

Metal–organic frameworks (MOFs) are a versatile class of porous crystalline materials composed of metal cations and organic linkers.^17–20^ The discovery of MOFs was recognized with the 2025 Nobel Prize in chemistry, awarded to Yaghi,^21^ Robson,^22^ and Kitagawa^23^ for pioneering the design and development of these coordination frameworks. The rapid development of MOF-based nanoparticles led to extensive applications, for example, as therapeutic agents,^17,24^ components of sensors,^25,26^ catalysts,^27^ and adsorbents.^28,29^ Together with the remarkable structural stability and catalytic efficiency, MOFs possess high porosity, a large number of active sites, and tunable morphology, making them fascinating nanozymes, while the presence of metal nodes (e.g., Cu, Fe, and Mn) within the structures endows them with intrinsic enzyme-mimicking capabilities.^8,30–32^ Despite these advances, the development of single-particle multifunctional (i.e., possessing more than one enzymatic function) MOF nanozymes that combine high biocatalytic versatility with advantageous structural features remains a rarely reported target.^24^

Bimetallic MOF-818 has been proven to function as an enzyme-mimetic nanomaterial due to the presence of redox active metal ions in the lattice. For instance, excellent oxidase-like activity was observed for a MOF-818 nanozyme via its trinuclear Cu centers,^33^ and effective mimicry of superoxide dismutase (SOD),^34^ catalase,^35^ and peroxidase (POD)^36^ enzymes was observed for similar nanomaterials indicating broad-spectrum antioxidant activity. Furthermore, in a pioneering work, sequential hydrolase- and oxidase-like activities were reported by jointly coordinating Ce and Cu in the MOF-818 structure.^37^ Despite the handful of reports on this topic, the development of multifunctional MOF-818 nanozymes is still a challenge. In addition, the colloidal behavior has not been studied systematically for MOF-818, and hence, the resistance of these nanozymes to aggregation under various dispersion conditions has yet to be elucidated, which is essential for designing efficient biocatalytic systems for application in aqueous dispersions.

Motivated by these gaps, we explore in this work both the multifunctional enzyme-mimetic behavior and the colloidal properties of solvothermally synthesized MOF-818 (CuZr-MOF). The investigated enzyme-like activities included SOD and POD mimics as an antioxidant feature followed by testing the protease-like function of the nanomaterial. Furthermore, the dispersion features, including hydrodynamic size and surface charge, have been systematically explored in various electrolyte solutions to probe the resistance of MOF-818 to salt-induced aggregation. Together, these results not only reveal the combined antioxidant and protease-mimicking activity of CuZr-MOF but also provide essential colloidal stability data to support the future design and application of multifunctional MOF-based nanozymes to be applied in liquid media.

## Results and Discussion

### Structural Characterization

The CuZr-MOF nanozyme was prepared via a previously reported procedure with some modifications; see the Experimental Section for a detailed synthesis. In brief, our method includes an initial sonication-assisted dissolution of the precursor metal salts and ligand in *N*,*N*-dimethylformamide (DMF) followed by the addition of trifluoroacetic acid (TFA) under stirring at 100 °C. Then the mixture was transferred to a Teflon-lined autoclave and placed in a preheated oven. This two-step solvothermal process provides enhanced control over nucleation and crystallization, leading to improved colloidal stability and reproducible particle formation.^34^ The synthesis process is illustrated in Figure 1a.

**Figure 1:**
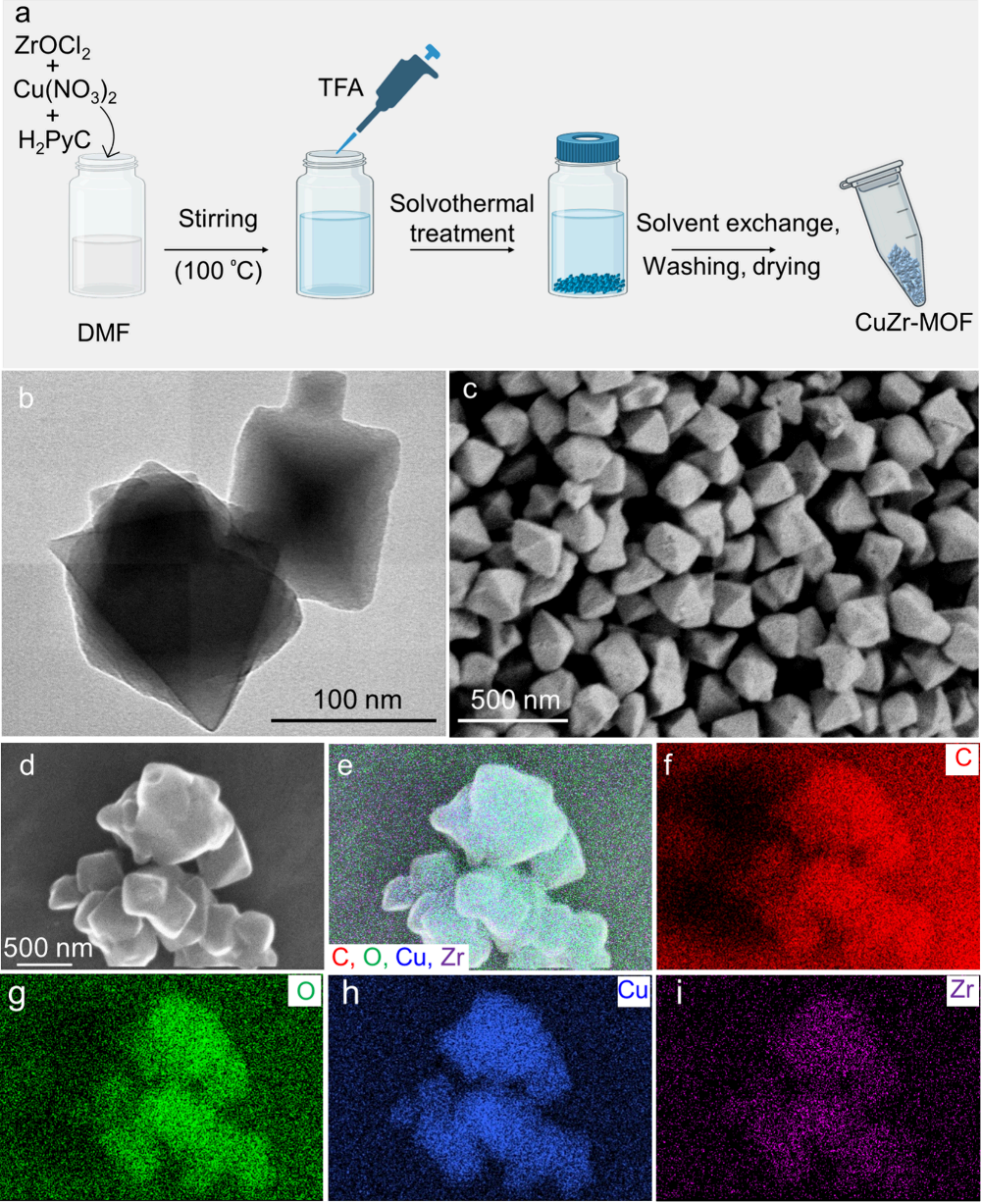
(a) Schematic illustration of the synthesis of the CuZr-MOF particles. (b) TEM and (c) SEM images of CuZr-MOF. (d–i) EDS mapping showing the elemental distributions. The scale bar in panel d applies to panels d–i.

Transmission electron microscopy (TEM) measurements were carried out to evaluate the morphology of the MOF-818. The images (for examples, see Figure 1b and Figure S1) revealed an octahedron shape for the particles with a uniform size of 186 ± 12 nm in diameter, which is somewhat smaller compared to previously reported MOFs,^33,34,36^ suggesting a higher specific surface area and enhanced dispersibility in liquid media. For further evaluation of the microstructure, the scanning electron microscopy (SEM) technique was applied. Figure 1c depicts the SEM image of the multizyme confirming uniformity in size and shape. The latter is similar to MOF-818 structures shown in earlier publications.^33^ EDS mapping (Figure 1d–i) demonstrates the homogeneous distribution of each element in the framework.

These results were further complemented with powder X-ray diffraction (PXRD) analysis (Figure 2a), which affirmed that the as-synthesized multizyme exhibited an F-centered cubic crystal lattice. The peaks located at 2.80°, 4.73°, 5.81°, and 6.71° correspond to the crystal structure of (111), (220), (222), and (400) planes, respectively. The diffraction peaks around 3° and 4°–8° indicate appropriate crystallinity of the CuZr-MOF and are in good agreement with the previous reports.^33,37^

**Figure 2:**
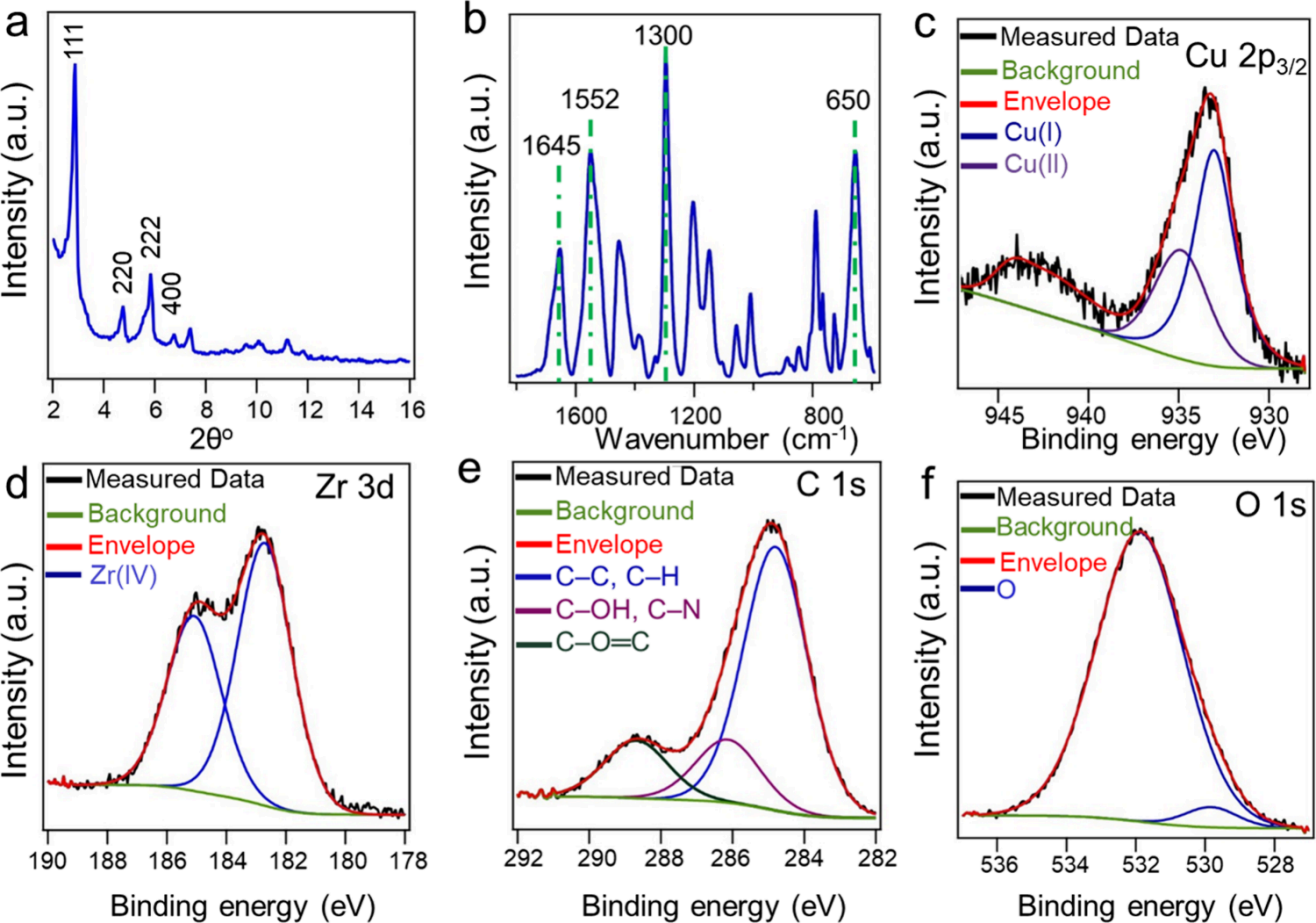
(a) Powder XRD pattern of the CuZr-MOF multizyme. The peaks with Miller indices characteristic of the MOF-818 compounds are indicated. (b) FTIR spectrum and (c–f) high-resolution core-level XPS spectra of CuZr-MOF.

Fourier transform infrared (FTIR) spectroscopy was employed for the identification of primary chemical bonds present in CuZr-MOF (Figure 2b and Figure S2a). In the wavenumber region (600–1800 cm^–1^) most characteristic of MOF compounds, various stretching and bending vibration bands could be identified. Accordingly, the strong absorption peak at 1645 cm^–1^ can be assigned to the symmetric stretching bands for C=O groups, whereas the sharp peaks in the 1552–1600 cm^–1^ range represent the asymmetric stretching of the O–C–O bond. Another intense peak at around 650 cm^–1^ corresponds to the O–Zr–O vibration. The bands between 1300 and 1110 cm^–1^ can be assigned to the presence of the C–N and C–O bonds in the CuZr-MOF including surface hydroxyl groups.^33,34,36^

The surface chemical composition of the CuZr-MOF was investigated by X-ray photoelectron spectroscopy (XPS). The appearance of peaks in the Cu 2p_3/2_ satellite region, i.e., 940–945 eV, confirms the presence Cu­(II) in the multizyme framework (Figure 2c). The additional peak at a lower binding energy of 933.04 eV corresponds to the existence of Cu­(I) and/or Cu(0) as it is difficult to distinguish between these oxidation states.^33^ Therefore, the Auger Cu LMM (electron transition levels, used to confirm oxidation state) spectrum was recorded to confirm the existence of Cu­(I) in the CuZr-MOF. As shown in Figure S2b, the Auger feature was found at 915 eV, which hints at the presence of Cu­(I), whereas no significant peak was observed around 918–920 eV, indicating that there is no Cu(0) in the MOF-818 framework.^33,35^ The peak in the Zr 3d region, between 182 and 186 eV, can be assigned to Zr­(IV), as shown in Figure 2d, while the peak at about 530 eV (Figure 2f) can be ascribed to the O 1s species. The spectra also suggest the presence of either surface hydroxyl groups or adsorbed water. Figure 2e displays the C 1s spectrum (deconvoluted into several characteristic peaks) indicating the presence of adventitious carbon on the sample surface.^33,34,36^ Figure S2c shows the full XPS survey of the multizyme. To gain insight into the elemental composition of the CuZr-MOF surface, quantitative analysis of the XPS results was carried out. As summarized in Table S1, the contributions of each element to the CuZr-MOF surface are determined to be 54.9%, 1.7%, 2.6%, 4.9%, 5.1%, 26.9%, and 3.9% for C, Cl, Cu, F, N, O, and Zr, respectively. Most of these elements include the structure of the compound prepared and charge compensating counterions, while a small amount of F can be attributed to residual TFA from the synthesis process. The XPS results also revealed a 49.4/50.6 surface ratio of Cu­(II)/Cu­(I) species (Table S2) responsible for the redox activity of the multizyme.

The integrated structural analysis verifies that the solvothermally synthesized CuZr-MOF exhibits the expected MOF-818 crystallinity, uniform particle morphology, and mixed-valence copper states essential for catalytic function.

### Colloidal Features

The dispersion properties including hydrodynamic radius and electrophoretic mobility (EM) of the MOF-818 were evaluated with dynamic (DLS) and electrophoretic (ELS) light scattering, respectively, at various pH values and electrolyte concentrations. First, the size and polydispersity index (PDI) were measured at different pH values (Figure 3a).

**Figure 3:**
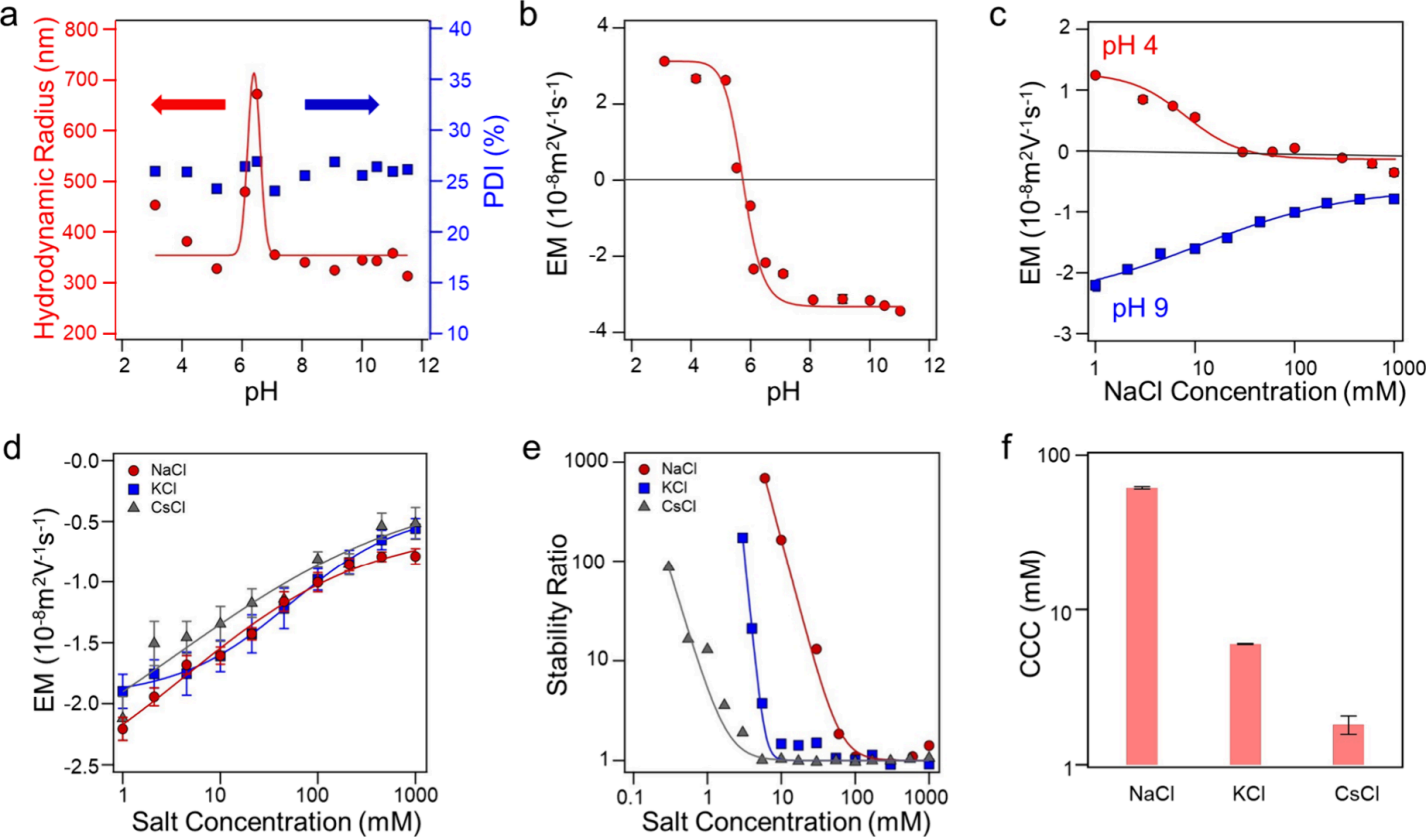
(a) Hydrodynamic radius, PDI, and (b) electrophoretic mobility (EM) of CuZr-MOF at various pH values. (c) EM versus NaCl concentration at pH 4 and 9 conditions. (d) Absolute value of EM and (e) stability ratio as a function of the concentration of NaCl, KCl, and CsCl. (f) CCC data of MOF-818 obtained in different electrolytes. The data shown in panels d–f were determined at pH 9, while all measurements were carried out at 10 mg/L particle concentration. The solid lines in panels a–d serve to guide the eye, while those in panel e represent the fits based on eq 2.

The trend in the hydrodynamic radii exhibited a maximum around pH 6, while the values remained between 300 and 350 nm at lower and higher pH. Note that the hydrodynamic size determined by DLS is larger compared to the one determined by electron microscopy. Such a deviation is due to the presence of trace aggregates in the dispersion, which contributes to the scattered intensity and shifts the mean hydrodynamic size toward larger values.^38^ The PDI data were approximately 25% in all samples, indicating moderate polydispersity in the size distribution of the CuZr-MOF particles. On the other hand, while investigating the EM in the same pH range, one can observe the isoelectric point (IEP) at pH 6 and that the particles carried a net positive charge at lower and negative charge at higher pH (Figure 3b). Such a pH-dependent surface charge is similar to the ones reported for inorganic materials and attributed to the protonation equilibria of the surface hydroxyl groups.^39^ Accordingly, the particle charge is determined by the presence of deprotonated or protonated hydroxyl functionalities, and the influence of structural metal ion composition and valence on the charge is negligible. Comparison between the tendencies in the pH dependence of the hydrodynamic radius (Figure 3a) and EM (Figure 3b) reveals the electrostatic origin of the stabilizing interparticle forces and that the size data can be qualitatively explained by the classical theory of Derjaguin, Landau, Verwey, and Overbeek (DLVO).^40,41^ Accordingly, the charged CuZr-MOF before and after the IEP is stabilized by electrical double layer repulsion and destabilized at the IEP due to the lack of such a double layer coupled with the dominance of attractive van der Waals forces. These latter interactions are responsible for particle aggregation and increased nanoparticle size around the IEP.

Since electrolytes are often present in applications of MOF materials in liquid media, the salt-dependent colloidal properties were studied. Figure 3c shows the EM of the obtained CuZr-MOF in NaCl solutions at pH 4 and 9. A consistency with previous data (Figure 3b) in terms of the sign of surface charge was observed at low salt levels, as positively and negatively charged CuZr-MOF particles were detected at these pH values, respectively. In general, the magnitude of the mobilities decreased by increasing the ionic strength due to the charge screening effect of the dissolved salts.^41^ However, such a decrease was more pronounced at pH 4, at which a slight charge reversal was also observed at higher NaCl concentrations, likely attributed to chloride counterion adsorption on the oppositely charged CuZr-MOF surface. At pH 9, the mobilities were negative throughout the entire NaCl concentration range investigated.

The colloidal properties presented above suggest that basic conditions, above the IEP, are more appropriate to further investigate aggregation processes in the CuZr-MOF dispersion. Therefore, pH 9 was chosen to study the possible effect of salt composition on the dispersion features by systematically varying the cation type, which are the counterions in the systems. The EM data in NaCl, KCl, and CsCl solutions at varying concentrations are shown in Figure 3d. No significant differences were detected in the EM obtained with NaCl and KCl, while the data were slightly less negative for CsCl, suggesting partial adsorption of Cs­(I) ions onto the CuZr-MOF surface.

To directly investigate the colloidal stability in these salt solutions, stability ratios (see eq 1) were determined under the same conditions as in the ELS study (Figure 3e). Note that the stability ratio is close to unity in rapidly aggregating dispersions (unstable samples), and higher values indicate slower aggregation (stable samples). Figure 3e shows that the stability ratios rapidly decrease with increasing salt concentration in all systems until they reach 1, which indicates fast aggregation of CuZr-MOF at higher salt concentrations. This tendency coincides with the predictions of the DLVO theory, and the salt concentration on the boundary between these two regimes is named the critical coagulation concentration (CCC). Note that the DLVO theory predicts the transition between slow and fast aggregation, but the difference in the CCC data caused by varying the chemical composition of the monovalent electrolyte cannot be explained, as this model considers point-like charges as ions and, hence, the same CCC for all monovalent electrolytes. For CuZr-MOF, the CCC values decrease in the NaCl > KCl > CsCl order (Figure 3f). Such ion specific effects on interparticle forces^42^ and subsequently on the aggregation rates and stability ratios^43^ can be explained by the Hofmeister series of ions.^44^ Accordingly, larger and less hydrated ions such as Cs­(I) adsorb more strongly to oppositely charged hydrophobic surfaces, while smaller and more hydrated ions (e.g., sodium) prefer to stay in the bulk solution. This is in line with the EM data for the CsCl and NaCl systems discussed earlier (Figure 3d). Stronger adsorption leads to a decrease in surface charge and weaker electrical double layer repulsion, and finally, lower salt concentration can induce particle aggregation compared to weakly adsorbing ions. Similar ion specific effects were observed with colloidal particles;^43,45^ however, these have never been reported for MOF compounds so far.

### Enzyme-Mimicking Ability

The enzyme-like function of the CuZr-MOF was assessed in terms of antioxidant and hydrolytic activities. First, the SOD-mimicking ability was determined with the modified Fridovich assay.^46^ Accordingly, the capability of the particles to scavenge superoxide radical anions resulting from the reaction of xanthine and xanthine oxidase was evaluated. The inhibition of the nitro blue tetrazolium (NBT)-radical reaction was calculated with eq 3 and shown as a function of particle concentration in Figure 4a.

**Figure 4:**
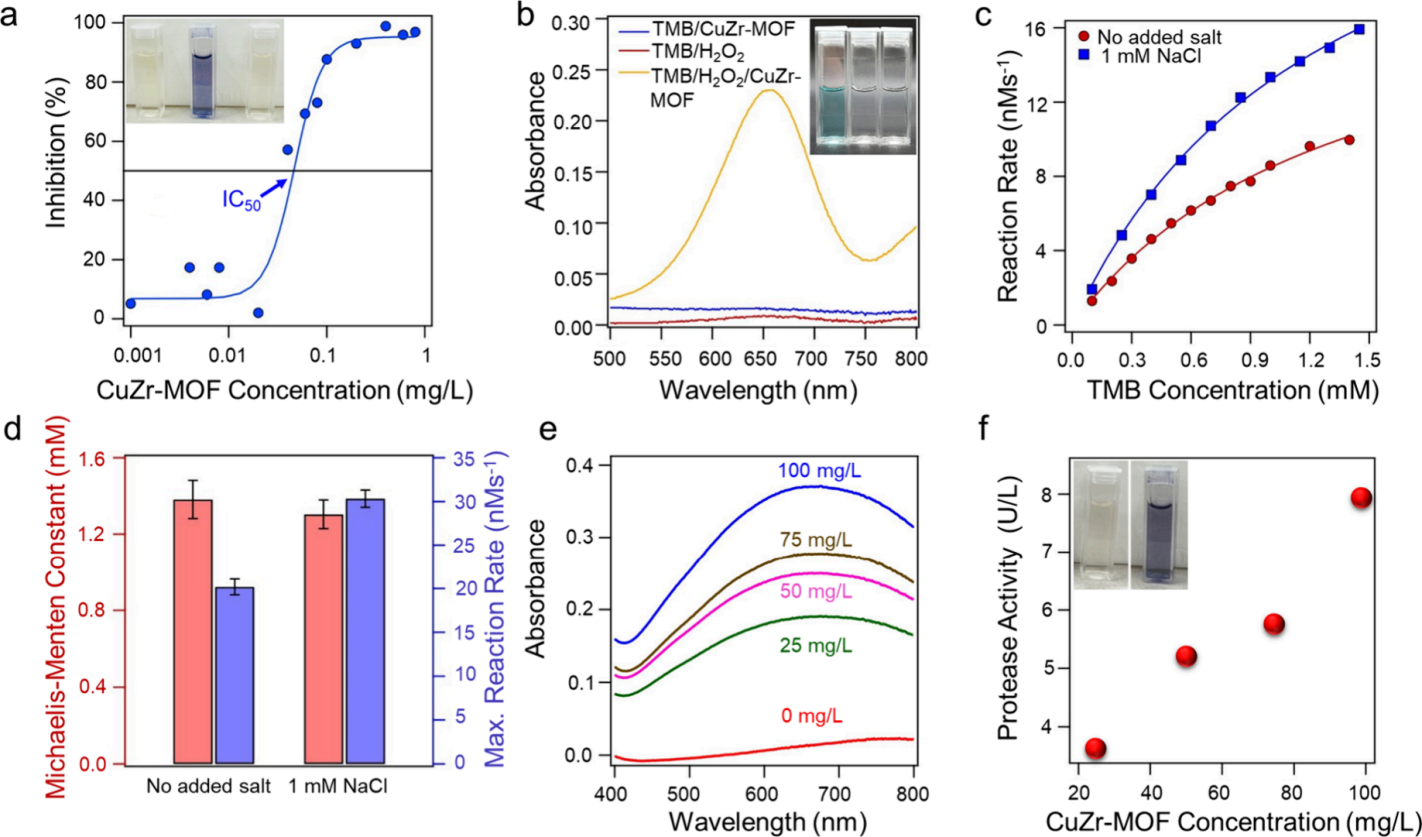
Nanozyme-like activities of the CuZr-MOF multizyme. (a) SOD-like activity with the color changes during the test illustrated in the inset (left: sample before the test, middle: sample after the test without CuZr-MOF, right: sample after the test with CuZr-MOF). (b) Absorbance peak measured during TMB oxidation (inset depicts the samples from left to right with CuZr-MOF/H_2_O_2_/TMB, TMB/H_2_O_2_, and CuZr-MOF). (c) Reaction rate of the TMB oxidation at varying substrate concentrations. (d) Michaelis–Menten constant and maximum reaction rate data. (e) UV–vis spectra for casein oxidation and (f) tyrosine production at various multizyme concentrations. In the latter, the inset shows the samples without (left) and with (right) CuZr-MOF.

The CuZr-MOF exhibited remarkable SOD-like activity with an IC_50_ value of 0.06 mg/L, which can be attributed to the small particle size and subsequent enlarged surface area for the biocatalytic reaction. This feature facilitates the transport of superoxide radicals to the active sites, i.e., Cu­(II) nodes in the nanozyme,^34^ which is responsible for the radical scavenge catalyzed by CuZr-MOF. Moreover, the near equimolar Cu­(II)/Cu­(I) ratio (Table S2) is advantageous, as both oxidation states participate in radical scavenging cycles. The developed nanoparticles exhibited excellent storage stability for more than 4 months. During these experiments, the aqueous dispersion of the multizyme was stored in a refrigerator at 4 °C, and it showed comparable SOD-like activity (Figure S3). Similar storage stability was reported for other antioxidant nanozyme systems reported earlier,^35^ indicating the improved resistance against time- or temperature-induced deactivation, which is one of the disadvantages of natural enzymes.

The horseradish peroxidase (HRP) assay was used to probe the POD-like function of CuZr-MOF.^47^ This test is based on the oxidation of the 3,3′,5,5′-tetramethylbenzidine (TMB) substrate in the presence of H_2_O_2_ via native HRP or its mimetic nanozyme.^47^ Figure 4b displays the HRP-like activity of the CuZr-MOF, in which the characteristic peak of oxidized TMB is observed around 652 nm. To run control experiments, TMB/H_2_O_2_ and TMB/CuZr-MOF combinations were measured under the same reaction protocol, and no significant absorbance peak was observed in these cases. Note that for the TMB/H_2_O_2_ combination, a small peak was observed at 652 nm showing the oxidation of a minor amount of TMB. This small peak was probably caused by the slow decomposition of H_2_O_2_ into hydroxyl radicals that led to the oxidation of TMB. The inset in Figure 4b depicts visual confirmation of TMB oxidation in the cuvette containing the blue sample in the presence of both H_2_O_2_ and CuZr-MOF, while no significant color change can be observed in the other tests. Figure 4c shows the reaction rates at varying TMB concentrations and fixed doses of CuZr-MOF (10 mg/L) and H_2_O_2_ (5 mM). Moreover, the HRP-like activity of the nanomaterial without added salt and in the presence of 1 mM NaCl is compared. Based on the increased reaction rates, it can be concluded that the notable activity is further enhanced by adding NaCl at a low concentration. This effect may be explained by the reduced thickness of the electric double layer upon addition of an electrolyte, which could make the nanozyme surface more accessible to the TMB substrates. Similar interpretation was reported in another nanozyme system, in which the concentration and valence of electrolytes were systematically varied in the samples.^16^ The data were interpreted in terms of the Michaelis–Menten model,^48^ and the fitting parameters (eq 3) are summarized in Figure 4d. No significant differences were observed in the Michaelis–Menten constants, while a remarkable increase in the maximum reaction rate was obtained in the presence of NaCl, indicating the improvement of the HRP-like function in the presence of the salt, as discussed above.

Note that despite the considerable SOD- and HRP-like activities discussed above, the CuZr-MOF particles did not show any catalase-like function, which was probed in H_2_O_2_ decomposition reactions.

In the next step, the possible protease-mimicking activity of CuZr-MOF was explored. Such a protein hydrolytic activity was investigated on the basis of the hydrolysis of casein, in which the formation of tyrosine was followed by spectrophotometry.^49^ Its reaction with the Folin–Ciocalteu reagent afforded a blue product, which was analyzed via its characteristic absorption peak at 660 nm to estimate the protease-like activity of the CuZr-MOF. Figure 4e depicts the measured absorbance data in the visible wavelength regime at various concentrations of the CuZr-MOF. The absorbance increases as particle concentration increases, indicating the catalytic hydrolysis of the casein substrate. Figure 4f shows the determined activity of CuZr-MOF in terms of tyrosine production at different multizyme concentrations, and the inset visually confirms the conversion of casein to tyrosine. Regarding the catalytic mechanism, it was assumed that the Zr­(IV) centers in the CuZr-MOF hydrolyze casein protein at the amide bonds, resulting in the release of amino acids including tyrosine. A plausible mechanism of protein hydrolysis is Lewis acidic activation of the water molecule and/or the carbonyl oxygen. In the CuZr-MOF, Zr­(IV) is a harder Lewis acid than Cu­(II), which makes it a more likely coordination site for oxygen (hard Lewis base) atoms. For example, Lewis acid catalyzed protein hydrolysis mechanisms for a natural metalloprotease were reported earlier, and its function was based on the presence of Zn­(II) ions in the enzyme,^50^ which is not redox active but rather a Lewis acidic center commonly found across bioinorganic chemistry. Although no direct experimental evidence could be obtained for this mechanism, results of detailed biocatalytic assays on a Zr­(IV)-based MOF material support the above scenario.^51^ The obtained protease-like activity value for CuZr-MOF was 24% of that determined for the papain enzyme (Figure S4). These data indicate a moderate function of the nanozyme compared to a native protease; however, its primary advantage lies in its multifunctionality, ease of preparation, moderate cost of storage, robustness, and long-term stability.

Based on the above findings, one can consider the CuZr-MOF a multizyme since SOD, HRP, and, for the first time, protease-like activities were all observed. During the dismutation of superoxide ions, its disproportionation occurs with the aid of Cu­(II) nodes, generating O_2_ and H_2_O_2_ as potential products. Subsequently, the active sites in the multizyme facilitate TMB oxidation, while the ROS component H_2_O_2_ concentration is reduced. In addition, the multizyme showed significant protease-like activity modeled by the breakdown of casein into amino acids such as tyrosine, thus greatly expanding its potential as an enzyme-mimicking nanomaterial. This multizyme is therefore a promising candidate for the development of stable and efficient nanozyme systems in a variety of areas including therapeutics, preservatives, and sensors.

## Conclusions

In conclusion, fine dispersions of CuZr-MOF multizymes were successfully synthesized via a solvothermal method, yielding uniform nanosized crystals with well-defined mixed-valence Cu centers essential for catalytic activity. A comprehensive colloidal analysis revealed that the particles exhibit predictable pH-dependent surface charging and tunable dispersion stability across a wide range of monovalent electrolyte concentrations. The observed ion specific trends, within the Hofmeister series of cations, demonstrated that counterion adsorption plays a decisive role in modulating the electrical double layer, aggregation kinetics, and critical coagulation concentrations of the MOF dispersions. These insights provide a solid physicochemical basis for optimizing the performance of MOF-based nanozymes in complex liquid environments.

The CuZr-MOF also displayed triple enzyme-mimicking activity, simultaneously functioning as a SOD, HRP, and, for the first time reported for MOF-818 compounds, a protease-like biocatalyst. This combined antioxidant and hydrolytic functionality is particularly noteworthy, as coapplication of natural antioxidant and protease enzymes is often hindered by mutual deactivation. In contrast, the structurally robust MOF lattice enables these activities to coexist within a single nanostructure without cross-interference. These findings underscore the potential of CuZr-MOF as a versatile multizyme platform capable of addressing biochemical challenges requiring concurrent ROS scavenging and controlled protein degradation. Beyond the specific CuZr-MOF system investigated here, the modular nature of the MOF architectures offers broad opportunities for further optimization. Systematic variation of metal ion types and oxidation state ratios, as well as surface functionalization strategies, can be exploited to tailor catalytic activity and aggregation processes. For instance, surface coating with polymeric compounds offers a promising route for extending stability under high ionic strength or complex medium conditions.

Altogether, the integration of biocatalytic versatility with well-known colloidal stability expands the scope for deploying MOF-type nanozymes in therapeutic formulations, bioprocessing, biosensing, and preservation technologies, particularly in aqueous dispersions. The structure–function relationships and interfacial behaviors revealed here provide a roadmap for the rational design of next-generation MOF multizymes with improved efficacy, selectivity, and operational robustness.

## Experimental Section

### Materials

1*H*-Pyrazole-4-carboxylic acid (H_2_PyC, 98%) was purchased from Apollo Scientific. TFA (99%) and zirconyl­(IV) chloride octahydrate (ZrOCl_2_·8H_2_O, 98%) were acquired from Thermo Fisher Scientific. Copper­(II) nitrate trihydrate (Cu­(NO_3_)_2_·3H_2_O, 99%) was purchased from Acros Organics. DMF (AnalaR NORMAPUR), H_2_O_2_ (30% m/m, AnalaR NORMAPUR), dimethyl sulfoxide (DMSO, analytical reagent), NaCl (GPR RECTAPUR), KCl (AnalaR NORMAPUR), CsCl (Ultra Pure), NaOH (AnalaR NORMAPUR), HCl (37% m/m, AnalaR NORMAPUR), nitro blue tetrazolium chloride monohydrate (NBT, ≥98%), sodium phosphate (monobasic, reagent grade), disodium phosphate (dibasic, reagent grade), and trichloroacetic acid (99.5%) were purchased from VWR. The water used throughout experiments was purified using an Adrona B30 water purification system and filtered through Millex VV filters (0.1 μm, Durapore PVDF membrane) to remove residual solid impurities.

### Synthesis of the Multizyme

The CuZr-MOF was prepared by the method reported previously^34^ with some modifications. Briefly, ZrOCl_2_·8H_2_O (85 mg), Cu­(NO_3_)_2_·3H_2_O (124 mg), and H_2_PyC (65 mg) were dissolved in 20 mL of DMF with the assistance of ultrasonic treatment (5 min), and then TFA (240 μL) was added to the solution. The mixture was heated at 100 °C under continuous stirring, transferred to a Teflon-lined autoclave (100 mL), and placed in a preheated oven for 10 h at 100 °C. The blue crystals were collected by centrifugation followed by the immersion in DMF, while changing the solvent after each 30 min. Then the product was washed three times with acetone and dried for 8 h at 80 °C. After the drying process, about 100 mg of light blue solid material was collected and stored at room temperature.

### Structural Characterization Methods

The morphology of CuZr-MOF was investigated using scanning electron microscopy (SEM, Thermo Scientific Apreo C) at an accelerating voltage of 10 kV and transmission electron microscopy (TEM, Jeol JEM-1400Plus) at 120 kV. The elemental distribution within the samples was determined by energy-dispersive X-ray spectroscopy (EDS) with a detector integrated into the SEM. Structural analysis was performed with X-ray diffraction (XRD) using a Philips PW 1830 diffractometer operating with a Cu anode (40 kV, 30 mA). The Cu Kα radiation was absorbed by a nickel filter. The patterns were recorded in the diffraction angle range 2°–16° with a resolution of 0.02° and scan rate of 50 s/°. The X-ray photoelectron spectroscopy (XPS) measurements were carried out with a SPECS instrument equipped with a PHOIBOS 150 MCD 9 hemispherical analyzer under a main-chamber pressure in the 10^9^–10^–10^ mBar range. The analyzer was in fixed transmission mode with a 40 eV pass energy for the survey scan and 20 eV for the high-resolution scan. To perform Fourier transform infrared spectroscopy (FTIR), a Nicolet Summit FTIR spectrometer (Nicolet Instrument Company) was employed using attenuated total reflectance (ATR) sampling.

### Light Scattering

The trends in the surface charge properties and hydrodynamic size of the CuZr-MOF were studied with electrophoretic (ELS) and dynamic (DLS) light scattering techniques, respectively. The electrophoretic mobility (EM) and hydrodynamic radius (*R*_h_) of the multizyme were recorded on an Anton Paar Litesizer 500 instrument equipped with a 658 nm laser source. When probing the pH dependence of colloidal properties, two 10 mg/L CuZr-MOF dispersions were prepared, and their pH was adjusted to 3 and 11, respectively. Afterward, a series of 5 mL samples in the pH range 3–11 were prepared by mixing different volumes of the two dispersions. Their exact pH values were measured with a WTW benchtop pH meter (inoLab pH 7310). During the salt-dependent ELS measurements, the concentration of the multizyme was kept at 10 mg/L in each sample, while the concentration of the salt was varied in the range of 1–1000 mM. The prepared dispersions were set aside to equilibrate for 2 h at room temperature before measurement. The 750 μL portions of the samples were used for ELS measurements performed at 25.0 ± 0.2 °C in Omega cuvettes (Anton Paar). EM values are reported as an average of five consecutive repetitions.

When probing the effect of pH, the *R*_h_ of the samples described above was also measured by DLS using the Litesizer 500 instrument at a scattering angle of 175°. The reported *R*_h_ values are the average of five consecutive runs. However, in the presence of monovalent salts, aggregation kinetics was evaluated using time-resolved DLS analysis (ALV/CGS-3 Compact Goniometer system) employing a 632.8 nm laser and a 90° scattering angle. The samples were prepared in borosilicate glass tubes (Kimble Chase), and the measurements were initiated immediately upon the addition of the nanozyme stock dispersion. For all samples, 100 measurement points were collected (20 s each), and the data were analyzed using the cumulant fit. Stability ratio (*W*) values were determined to express colloidal stability as^38,45^

$$W=\frac{(dR_{h}\left(t\right)/dt{)}_{t\to 0,\mathrm{fast}}}{(dR_{h}\left(t\right)/dt{)}_{t\to 0}}$$ 1

where *dR*_h_(*t*)/*dt* is the slope of the linear fit on the hydrodynamic radius-time (*t*) data points. The fast condition refers to 1.0 M ionic strength, where electrostatic forces are screened, and at this stage, the aggregation is mainly governed by the diffusion of the particles. The magnitude of the stability ratio reflects the colloidal stability of the corresponding dispersion. Samples undergoing fast aggregation have a stability ratio close to 1, whereas higher values indicate stable dispersions. The results were visualized using the stability ratio versus salt concentration plots, and the data points were fitted with the following equation to calculate the critical coagulation concentration (CCC) values as

$$W=1+{\left(\frac{\mathrm{CCC}}{c}\right)}^{-\beta }$$ 2

where *c* is the salt concentration and β is the slope determined in the slow aggregation regime before the CCC.

### Enzyme Assays

The Fridovich assay was employed to assess the SOD-like activity of the multizyme.^46^ The superoxide radicals in this assay were produced as a byproduct of the oxidation of xanthine under the catalytic action of xanthine oxidase. The superoxide radical ions convert the yellow-colored NBT indicator into blue-colored diformazan. SOD-mimicking nanozymes inhibit this reaction by catalyzing the decomposition of the superoxide ions, thus preventing the formation of diformazan. The efficiency of the nanozyme is therefore related to the intensity of the blue color formed, which can be monitored by UV–vis spectrophotometry (Thermo Scientific GENESYS 10S). Typically, a series of 3000 μL samples were prepared, in which only the concentration of the multizyme was varied in the 0–1 mg/L range. All stock solutions, except for the multizyme dispersion, were prepared in 10 mM phosphate buffer (pH 7.0). Each sample contained 200 μL of 3.0 mM xanthine and 100 μL of 3.0 mM NBT as well as appropriate amounts of multizyme stock and 10 mM phosphate buffer to obtain a total volume of 2700 μL. The sample was homogenized, and then 300 μL of 1.5 g/L xanthine oxidase solution was added to initiate the reaction. The cuvette was vortexed and immediately introduced into the spectrophotometer, where the absorbance values were recorded at 565 nm for 6 min. In addition, blank samples (without CuZr-MOF) were also measured. The resulting inhibition (*I*) of the superoxide radical-NBT reaction can be obtained with the following equation as^15^

$$I=\frac{\Delta A_{0}-\Delta A_{s}}{\Delta A_{0}}\cdot 100$$ 3

where Δ*A*_0_ is the average value of 6 min absorbance change for the blank samples and Δ*A*_s_ is the change in absorbance during the 6 min measurement time for each sample. The concentration of the nanozyme at which 50% inhibition occurs is known as the IC_50_ value. It is noteworthy that light scattering by the multizyme has a contribution to the absorbance values during the assays. However, this effect was excluded by using only the relative increase in the absorbance in each test.

The HRP-like activity of the multizyme was assessed employing the HRP assay, which is based on the oxidation of TMB in the presence of H_2_O_2_ and HRP or its mimetic nanozyme.^47^ The oxidation of the colorless TMB affords a blue product, the presence of which can be confirmed by observing its characteristic peak at 652 nm via UV–vis spectrophotometry. Owing to the poor water solubility of TMB, stock solutions were prepared in DMSO, and its concentration was varied in the samples between 0 and 1.5 mM, while the concentrations of the multizyme and H_2_O_2_ were kept unchanged. Subsequently, a series of samples were prepared containing a varied volume of 10 mM TMB stock solution, 40 μL of 500 mg/L CuZr-MOF stock dispersion (set to pH 4 using HCl), and an appropriate volume of HCl solution (pH 4) to obtain a total volume of 1800 μL. Finally, 200 μL of 50 mM H_2_O_2_ stock solution (adjusted to pH 4) was added to obtain the final 2000 μL volume. The samples were briefly homogenized, and the absorbances at 652 nm were recorded over a period of 10 min. The slopes of the absorbance versus time plots were used to obtain the reaction rates (*v*), measured in 1/s units. The Beer–Lambert law can be used to convert these values to molar rate (M/s), considering the optical light path of 1 cm and the molar extinction coefficient of the oxidized TMB of 39,000 M^–1^ cm^–1^. Ultimately, the reaction rate was plotted against the concentration of TMB ([*S*]) followed by evaluating the kinetics of the multizyme catalyzed TMB oxidation. The Michaelis–Menten model was employed for this purpose, which can be expressed in the following equation as^48^

$$v=\frac{v_{\max}\left[S\right]}{K_{M}+\left[S\right]}$$ 4

where *v*_max_ is the maximum reaction rate approached at large [*S*] and *K*_M_ is the Michaelis–Menten constant. At a TMB concentration of *K*_M_, the reaction rate is equal to half of *v*_max_.

To evaluate the hydrolytic activity of the multizyme, a universal protease activity protocol by Sigma-Aldrich was used, which is based on the Lowry method with a little modification.^49^ Accordingly, a series of standard tyrosine solutions with varied concentrations were prepared from a 1.1 mM stock solution, as tyrosine serves as the product of the hydrolysis in the test reaction, and its concentration range in our experiment was 27–150 μM during the calibration. Different volumes of ultrapure water were added to adjust a final volume of 2 mL. The samples were incubated at 37 °C for 30 min. During the protease-like activity studies of CuZr-MOF, casein was selected as a model substrate, which is widely accepted in universal protease assays. In a typical experiment, 5 mL of 0.65 w/v% casein as a substrate was prepared using 50 mM phosphate buffer solution (pH 7.5) as the solvent. A varied volume of the multizyme or papain was added to 0.5 mL of casein followed by the addition of a known volume of buffer to adjust the volume to 1.1 mL. Then, the reaction took place for 10 min, and then 0.5 mL of 110 mM trichloroacetic acid was added to stop the hydrolysis process followed by incubation at 37 °C for 30 min. The samples were filtered with a 0.45 μm syringe filter (Millex), and 0.75 mL of 0.5 M sodium carbonate and 0.1 mL of 0.5 M Folin–Ciocalteu reagent were added to both series, i.e., standard and test samples, which were incubated again at 37 °C for 30 min. Finally, about 2 mL aliquots were centrifuged at 8000 rpm for 5 min, and absorbance values were recorded at 660 nm (see the calibration curve in Figure S5). One unit of hydrolytic activity is equivalent to the amount of multizyme required for 1 μg of tyrosine/mL/min to be released under the assay conditions described above.

## Supplementary Material



## Notes

TEM images of CuZr-MOF, elemental analysis data obtained by XPS, FTIR and XPS spectra of CuZr-MOF, SOD- and protease-like activity data of the multizyme and native enzyme, and calibration curve for tyrosine concentration determination (PDF)
