# Supplementary material for: Metal–Organic Framework Multizyme Colloids with Joint Antioxidant and Protease Function
Source: Langmuir. 2026 Jul 7;42(28):20104–13. doi: 10.1021/acs.langmuir.5c06847 (PMC13394421; doi:10.1021/acs.langmuir.5c06847)
Supplement: Supplementary file 1 [file la5c06847_si_001.pdf]

Supporting Information (SI)

Metal-Organic Framework Multi-Zyme Colloids with Joint Antioxidant and Protease Function

*Laila Noureen,[‡] Dániel Viczián,[‡] Gergely F. Samu,[ǂ] Imre Szenti,[§] Bojana Katana,[‡†] Tamás Szabó,[£] Viktoria Hornok,[‡] Zoltán Kónya,[§] and Istvan Szilagyi[‡]**

[‡]MTA-SZTE Momentum Biocolloids Research Group, Interdisciplinary Centre of Excellence, University of Szeged, 6720 Szeged, Hungary

[ǂ]Department of Molecular and Analytical Chemistry, University of Szeged, 6720 Szeged, Hungary

[§]Department of Applied and Environmental Chemistry, University of Szeged, 6720 Szeged, Hungary

[†]Institute of Condensed Matter and Nanosciences - Bio and Soft Matter, Université catholique de Louvain, 1348 Louvain-la-Neuve, Belgium

[£]Department of Physical Chemistry and Materials Science, University of Szeged, 6720 Szeged, Hungary

*Corresponding author. E-mail: szistvan@chem.u-szeged.hu

Table S1. Quantitative XPS analysis for the elements present in CuZr-MOF.

**Element**	**Atomic Percentage (%)**
Carbon	54.9
Chloride	1.7
Copper	2.6
Fluoride	4.9
Nitrogen	5.1
Oxygen	26.9
Zirconium	3.9

Table S2. Quantitative XPS analysis for the existence of various ionic species in CuZr-MOF.

**Species**	**Percentage (%)**
Cu(I)	49.4
Cu(II)	50.6
Cu-N	74.0
Pyridazole	26.0

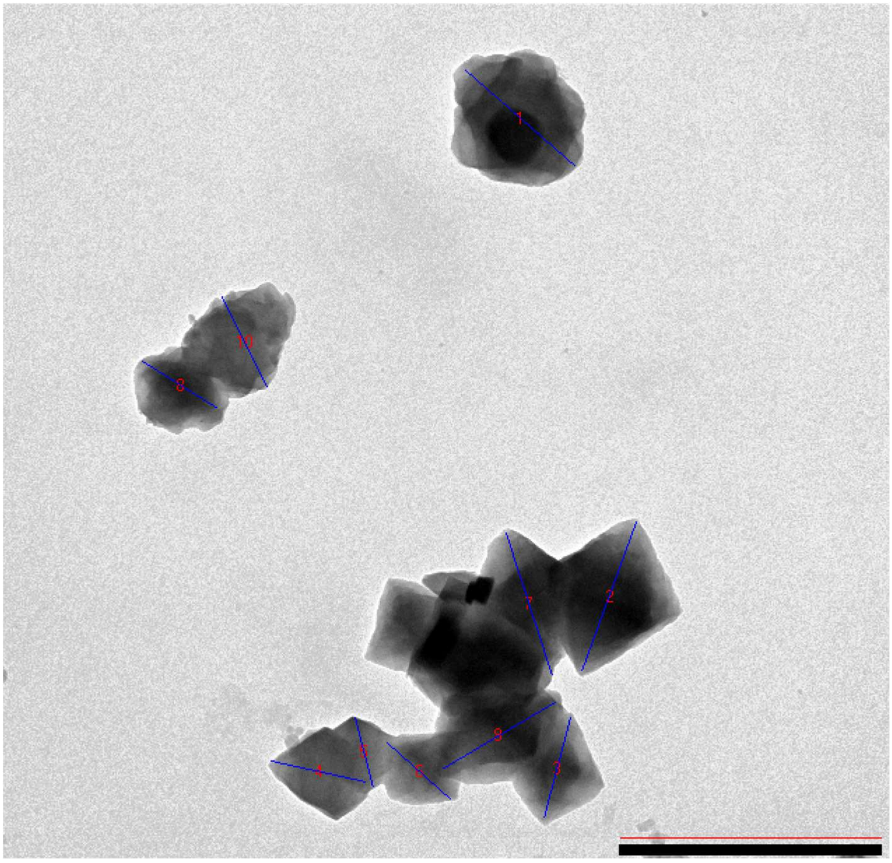

Figure S1. Illustrative TEM image of the CuZr-MOF nanozymes. The particle size was estimated based on TEM images using the Nanosize measurer 1.2.5 software. The scale bar is 500 nm and red line of the same length was used for calibration. The average particle size was found to be 186 ± 12 nm in diameter. Note that blue lines across particles are measurement markers drawn with the above software.

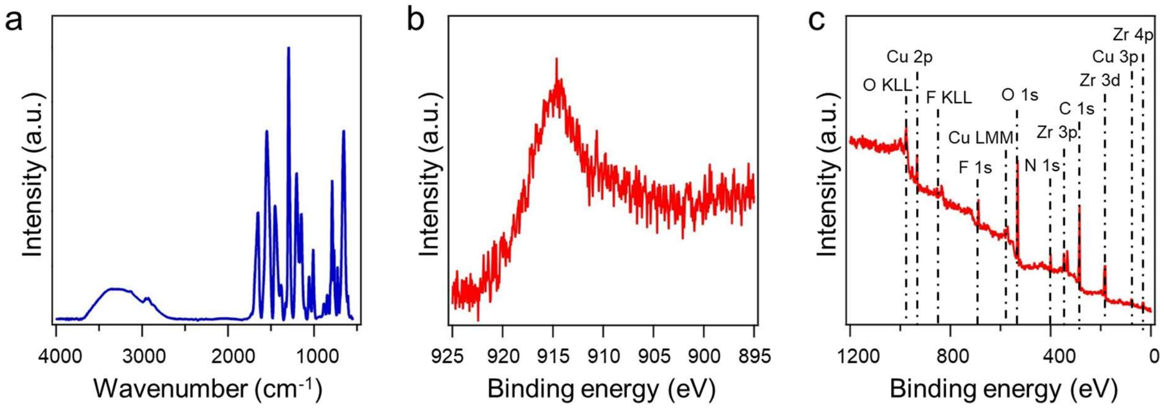

Figure S2. a) FT-IR spectrum, b) Cu Auger LMM and c) XPS full survey of the CuZr-MOF multi-zyme.

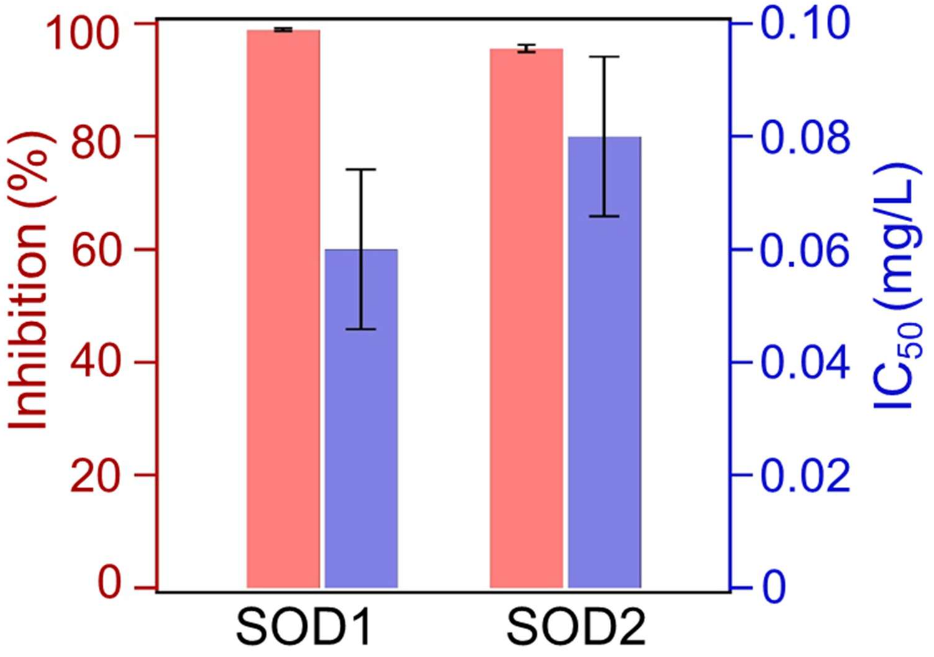

Figure S3. Storage stability of CuZr-MOF at 4 °C. SOD-like activity of the multi-zyme expressed in inhibition of radical-NBT reaction (first columns) as well as in IC_{50} data (second columns) with freshly prepared samples (SOD1) and after 4 months (SOD2).

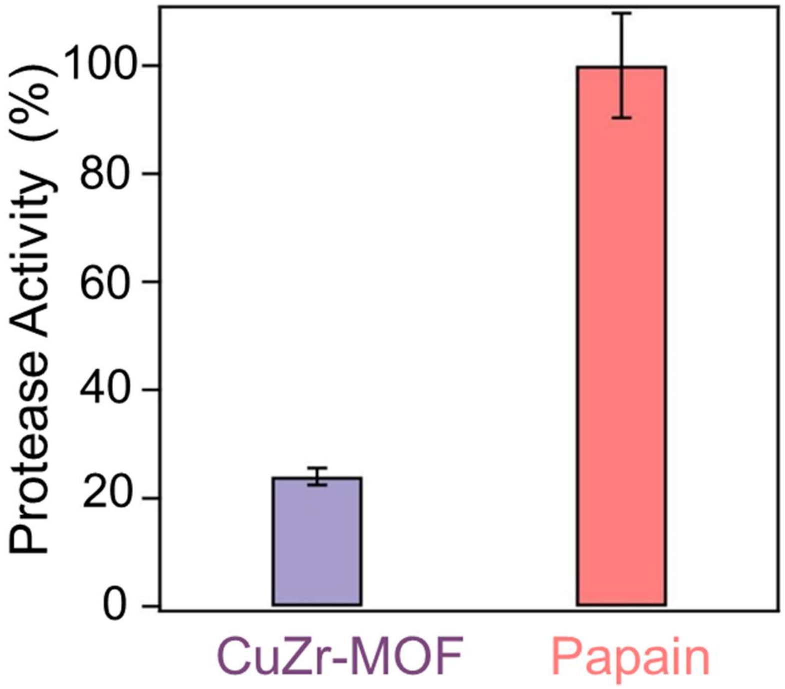

Figure S4. Protease-mimicking activity of CuZr-MOF versus natural protease enzyme papain. The tests were evaluated and compared with papain under identical conditions, where activity of the native enzyme was designated as 100%, while pristine CuZr-MOF showed lower, but measurable and reproducible protease-like activity.

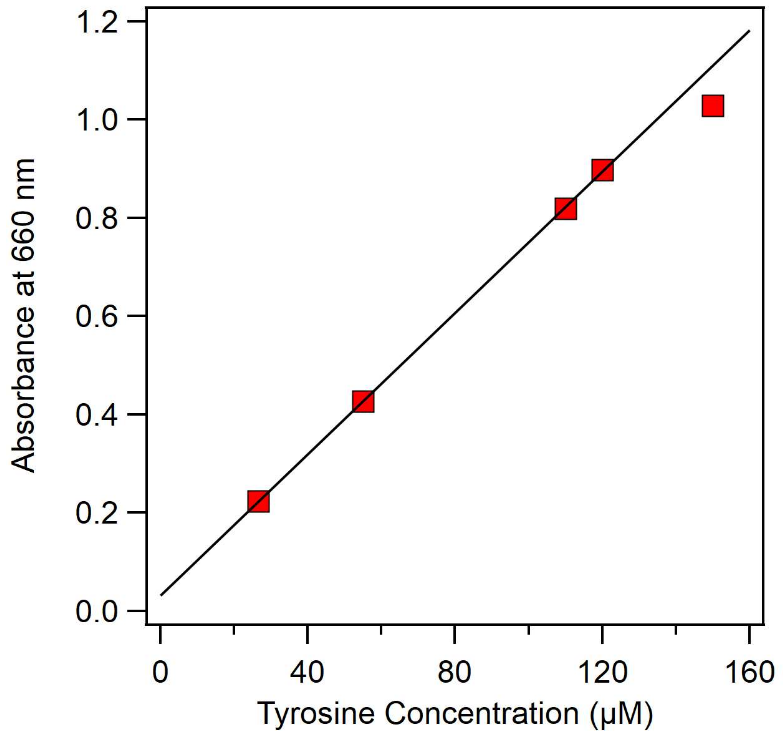

Figure S5. Absorbance read at 660 nm versus the tyrosine concentration. The calibration curve was used for determination of protease activity in the linear regime up to 120 μM concentration of tyrosine.
